# Longitudinal Assessment of Seasonal Impacts and Depression Associations on Circadian Rhythm Using Multimodal Wearable Sensing: Retrospective Analysis

**DOI:** 10.2196/55302

**Published:** 2024-06-28

**Authors:** Yuezhou Zhang, Amos A Folarin, Shaoxiong Sun, Nicholas Cummins, Yatharth Ranjan, Zulqarnain Rashid, Callum Stewart, Pauline Conde, Heet Sankesara, Petroula Laiou, Faith Matcham, Katie M White, Carolin Oetzmann, Femke Lamers, Sara Siddi, Sara Simblett, Srinivasan Vairavan, Inez Myin-Germeys, David C Mohr, Til Wykes, Josep Maria Haro, Peter Annas, Brenda WJH Penninx, Vaibhav A Narayan, Matthew Hotopf, Richard JB Dobson, RADAR-CNS consortium

**Affiliations:** 1 Department of Biostatistics & Health Informatics Institute of Psychiatry, Psychology and Neuroscience King's College London London United Kingdom; 2 Institute of Health Informatics University College London London United Kingdom; 3 NIHR Biomedical Research Centre at South London and Maudsley NHS Foundation Trust London United Kingdom; 4 NIHR Biomedical Research Centre at University College London Hospitals, NHS Foundation Trust London United Kingdom; 5 Health Data Research UK London University College London London United Kingdom; 6 Department of Computer Science University of Sheffield Sheffield United Kingdom; 7 Department of Psychological Medicine Institute of Psychiatry, Psychology and Neuroscience King's College London London United Kingdom; 8 School of Psychology University of Sussex Falmer United Kingdom; 9 Department of Psychiatry Amsterdam UMC Vrije Universiteit Amsterdam Netherlands; 10 Mental Health Program Amsterdam Public Health Research Institute Amsterdam Netherlands; 11 Centro de Investigación Biomédica en Red de Salud Mental Madrid Spain; 12 Teaching Research and Innovation Unit Parc Sanitari Sant Joan de Déu Fundació Sant Joan de Déu Barcelona Spain; 13 Faculty of Medicine and Health Sciences Universitat de Barcelona Barcelona Spain; 14 Department of Psychology Institute of Psychiatry, Psychology and Neuroscience King's College London London United Kingdom; 15 Janssen Research and Development LLC Titusville, NJ United States; 16 Department of Neurosciences Center for Contextual Psychiatry Katholieke Universiteit Leuven Leuven Belgium; 17 Center for Behavioral Intervention Technologies Department of Preventive Medicine Northwestern University Chicago, IL United States; 18 South London and Maudsley NHS Foundation Trust London United Kingdom; 19 H Lundbeck A/S Copenhagen Denmark; 20 Davos Alzheimer’s Collaborative Geneva Switzerland; 21 See Acknowledgments

**Keywords:** circadian rhythm, biological rhythms, mental health, major depressive disorder, MDD, wearable, mHealth, mobile health, digital health, monitoring

## Abstract

**Background:**

Previous mobile health (mHealth) studies have revealed significant links between depression and circadian rhythm features measured via wearables. However, the comprehensive impact of seasonal variations was not fully considered in these studies, potentially biasing interpretations in real-world settings.

**Objective:**

This study aims to explore the associations between depression severity and wearable-measured circadian rhythms while accounting for seasonal impacts.

**Methods:**

Data were sourced from a large longitudinal mHealth study, wherein participants’ depression severity was assessed biweekly using the 8-item Patient Health Questionnaire (PHQ-8), and participants’ behaviors, including sleep, step count, and heart rate (HR), were tracked via Fitbit devices for up to 2 years. We extracted 12 circadian rhythm features from the 14-day Fitbit data preceding each PHQ-8 assessment, including cosinor variables, such as HR peak timing (HR acrophase), and nonparametric features, such as the onset of the most active continuous 10-hour period (M10 onset). To investigate the association between depression severity and circadian rhythms while also assessing the seasonal impacts, we used three nested linear mixed-effects models for each circadian rhythm feature: (1) incorporating the PHQ-8 score as an independent variable, (2) adding seasonality, and (3) adding an interaction term between season and the PHQ-8 score.

**Results:**

Analyzing 10,018 PHQ-8 records alongside Fitbit data from 543 participants (n=414, 76.2% female; median age 48, IQR 32-58 years), we found that after adjusting for seasonal effects, higher PHQ-8 scores were associated with reduced daily steps (β=–93.61, *P*<.001), increased sleep variability (β=0.96, *P*<.001), and delayed circadian rhythms (ie, sleep onset: β=0.55, *P*=.001; sleep offset: β=1.12, *P*<.001; M10 onset: β=0.73, *P*=.003; HR acrophase: β=0.71, *P*=.001). Notably, the negative association with daily steps was more pronounced in spring (β of PHQ-8 × spring = –31.51, *P*=.002) and summer (β of PHQ-8 × summer = –42.61, *P*<.001) compared with winter. Additionally, the significant correlation with delayed M10 onset was observed solely in summer (β of PHQ-8 × summer = 1.06, *P=*.008). Moreover, compared with winter, participants experienced a shorter sleep duration by 16.6 minutes, an increase in daily steps by 394.5, a delay in M10 onset by 20.5 minutes, and a delay in HR peak time by 67.9 minutes during summer.

**Conclusions:**

Our findings highlight significant seasonal influences on human circadian rhythms and their associations with depression, underscoring the importance of considering seasonal variations in mHealth research for real-world applications. This study also indicates the potential of wearable-measured circadian rhythms as digital biomarkers for depression.

## Introduction

Depression is a globally prevalent mental disorder with various negative impacts, including reduced quality of life, disability, premature mortality, and an increased risk of suicide [1-6]. However, current depression diagnostic methods face several critical limitations: (1) reliance on individuals’ subjective recall of past behaviors, which introduces recall bias and neglects day-to-day fluctuations [7,8]; (2) dependence on skilled and experienced clinicians [9,10]; and (3) evaluations often delayed until the mental health issues have progressed to a more severe, difficult-to-treat stage [11]. Consequently, these limitations result in the underdiagnosis and delayed treatment for those with depression [12,13], underscoring the critical need for objective and timely methods for early detection.

Previous research has found significant links between circadian rhythms and depression [14,15]. Circadian rhythms are approximately 24-hour endogenous oscillations, controlled by the master clock in the suprachiasmatic nucleus of the hypothalamus, that regulate many aspects of human behavior and physiology, such as sleep-wake cycles, hormone secretion, and body temperature [16-18]. Disturbances in circadian rhythms have been associated with an increased risk of both physical and mental diseases [15,19-21]. Therefore, tracking human circadian rhythms is a potential objective method for early-stage depression identification. Traditional methods for circadian rhythm assessment involve tracking melatonin in bodily fluids (such as saliva, urine, or blood samples) in a constant light environment to prevent external light from altering melatonin production and biasing the assessment of the circadian phase [22,23]. However, this traditional approach is expensive, labor-intensive, and impractical for large cohort studies and long-term monitoring in real-world settings [24,25].

With the development of ubiquitous sensors and mobile technologies, wearable devices provide a convenient and cost-effective way to continuously monitor individuals’ daily behaviors and physiological signals in real-world settings [26]. Previous mobile health (mHealth) studies have explored the approximation of human circadian rhythms through wearable-measured patterns, including sleep-wake cycles, rest-activity patterns, and circadian rhythms in heart rate (HR) [24,27-32]. Higher depression severity has been found to be associated with later sleep onset and offset times, higher sleep variability, lower amplitude of activity, lower intradaily stability, and later acrophase of activity and HR [24,27-32].

However, previous mHealth studies have not fully accounted for seasonal variations, possibly due to their short study durations. Seasonal changes in sunlight and temperature are crucial environmental zeitgebers for the internal circadian clock, impacting human circadian rhythms [33-35]. Prior research has reported significant seasonal effects on sleep patterns and activity levels [36-39]. Ignoring these seasonal impacts may introduce bias into the associations between depression and wearable-measured circadian rhythms in real-world settings. Thus, examining the effects of seasonality on wearable-measured circadian rhythms and their connections to depression in a comprehensive longitudinal data set is needed.

The primary aim of this study was to explore the associations between depression severity and wearable-measured circadian rhythms, accounting for seasonal effects and investigating potential variations across seasons. Our secondary aim was to quantify the seasonal changes in wearable-measured circadian rhythms within a European mHealth study for depression [40].

## Methods

### Data Set

#### Participants and Settings

The data analyzed in this study were sourced from the Remote Assessment of Disease and Relapse Major Depressive Disorder (RADAR-MDD) research program, which aimed to investigate the utility of remote technologies for monitoring depression and understanding factors that could help predict relapse in MDD [40]. A total of 623 participants were recruited from 3 study sites across 3 European countries (United Kingdom, Spain, and the Netherlands) and followed for up to 2 years [41]. The first participant was enrolled in November 2017 and the last participant was enrolled in June 2020, and the data collection was completed in April 2021. Due to this rolling enrollment process, participants’ involvement in the study varied from 11 months to 24 months [41]. The RADAR-MDD program used the RADAR-base open-source platform to concurrently gather both active (eg, questionnaires) and passive (eg, wearable) data [42]. Comprehensive details of the study protocol and data set have been documented in publications [40] and [41], respectively.

#### Patient Involvement

The RADAR-MDD protocol was co-developed with a patient advisory board who shared their opinions on several user-facing aspects of the study including the choice and frequency of survey measures, the usability of the study app, participant-facing documents, selection of optimal participation incentives, selection and deployment of wearable devices, and the data analysis plan.

#### Ethical Considerations

Ethical approvals were obtained from the Camberwell St. Giles Research Ethics Committee (17/LO/1154) in the United Kingdom, the Fundacio Sant Joan de Deu Clinical Research Ethics Committee (CI: PIC-128-17) in Spain, and the Medische Ethische Toetsingscommissie VUmc (2018.012- NL63557.029.17) in the Netherlands. All participants provided their written informed consent. Before the data collection, participants were assured of their privacy protection, informed of their right to withdraw at any time without needing to justify their decision, and allowed to request the deletion of all their collected data. The data from participants were pseudonymized and stored in a research database, adhering to the General Data Protection Regulation. Participants were compensated with £15 (US $19.09)/€20 (US $21.68) for enrollment, £5 (US $6.36)/€10 (US $10.84) for each clinical assessment conducted every 3 months, and an additional £10 (US $12.73)/€10 (US $10.84) for each qualitative interview they completed. No participant is identifiable in any images in the manuscript or the supplementary materials. Further details can be found in the study protocol and data description papers [40,41].

### Measures

#### Depression Symptom Severity

Participants’ depression symptom severity was measured using the 8-item Patient Health Questionnaire (PHQ-8) [43] conducted via mobile phones every 2 weeks. The PHQ-8 comprises 8 questions, and the total score of PHQ-8 ranges from 0 to 24, indicating increasing severity [43].

#### Fitbit Data

Participants were asked to wear a Fitbit Charge 2/3 wrist-worn device throughout the whole study. Participants’ sleep, step count, and HR were continuously (24/7) measured and recorded.

- Sleep data: Fitbit provided sleep labels (“awake,” “light sleep,” “deep sleep,” and “rapid eye movement”) along with the corresponding local clock times every 30 seconds.
- Step data: Participants’ accumulated steps were counted every minute.
- HR data: Fitbit provided an estimate of HR every 5 seconds, using an embedded photoplethysmography sensor. However, technical issues resulted in the absence of some sample points. To obtain the robust HR trend and align with step data, we calculated the average HR over 1 minute.

#### Season

The seasonal division used in this study was based on European Union astronomical seasons: spring (March 20 to June 20), summer (June 21 to September 22), autumn (September 23 to December 20), and winter (December 21 to March 19).

#### Covariates

In accordance with findings from previous studies [44-46], we considered several covariates that could potentially influence participants’ circadian rhythms, including age, gender, and employment status. Because the COVID-19 pandemic and relevant restrictions had some significant impacts on individuals’ behavior [47], we introduced a covariate “lockdown” to indicate the presence of a national lockdown. Furthermore, as the experience of seasons can be different across countries, the study site was also considered as a covariate. These covariates were considered in our statistical analysis.

### Feature Extraction

#### PHQ-8 Interval

**Figure 1:**
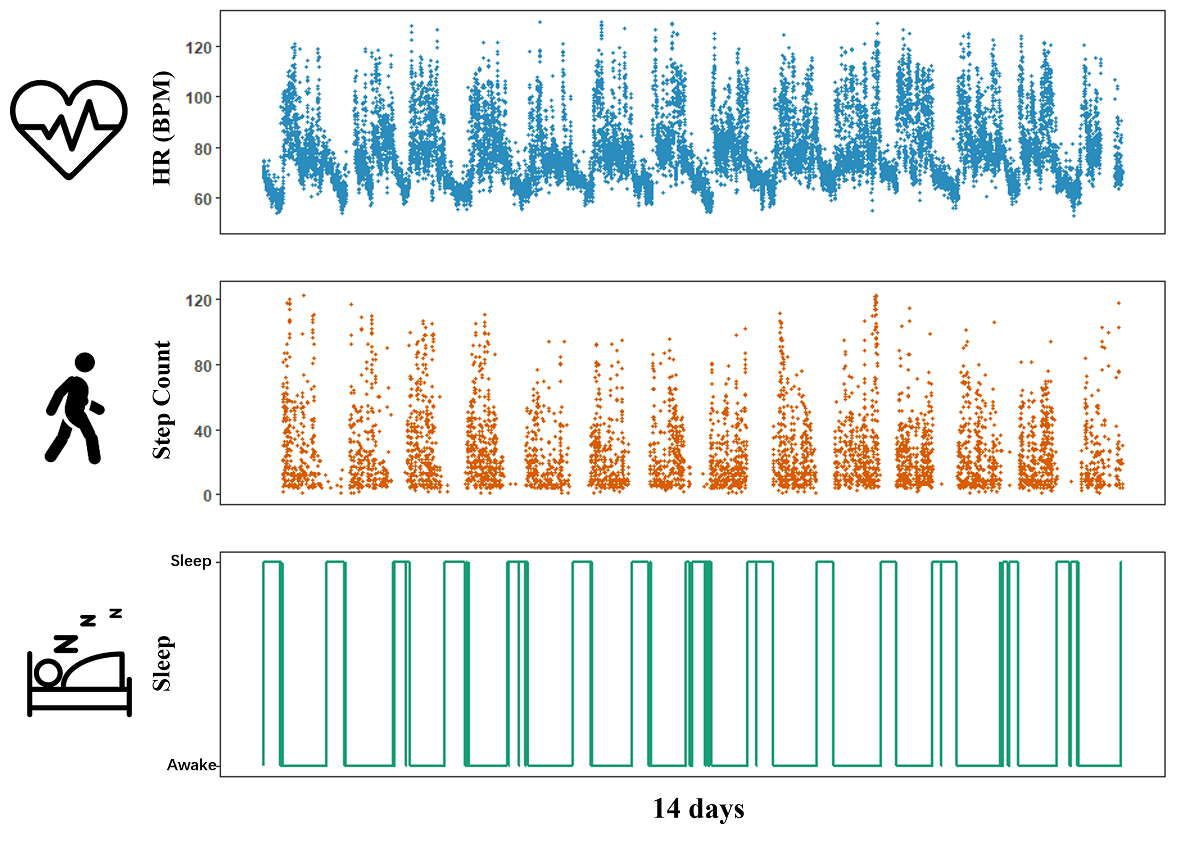
An example of a participant’s processed heart rate (HR), step, and sleep Fitbit data during the preceding 14 days of an 8-item Patient Health Questionnaire assessment collected via the RADAR–base platform.

To link human circadian rhythms with depression severity, we extracted circadian rhythm features from each 14-day PHQ-8 interval—specifically, 14 days of Fitbit data preceding a completed PHQ-8. This feature window aligns with the PHQ-8’s purpose of evaluating depressive symptom severity over the past 2 weeks [43] and is consistent with both the existing literature in the mHealth field [48-50] and our previous studies [51,52]. Figure 1 shows an example of a participant’s processed HR, step, and sleep data in a 14-day PHQ-8 interval.

#### Data Inclusion Criteria

Some Fitbit data were missing in our data set for several reasons, including device damage, low battery level, and not being worn. Building on insights from our prior research [53], which discussed the sufficient days for stable feature calculation, we focused on 14-day PHQ-8 intervals with at least 8 days having more than 80% of step and HR data and a sleep recording in this study. Considering the potential impact of daylight saving time on individuals’ behaviors [54], we excluded the first 14-day PHQ-8 interval after the time switching [37]. We then extracted a total of 12 features for reflecting the circadian rhythms from sleep, step count, and HR data.

#### Sleep-Wake Rhythms

Fitbit shows promise in identifying sleep-wake status [55,56]. Therefore, to reflect the sleep-wake rhythms, we computed four features: (1) sleep duration (the mean total sleep time), (2) sleep variability (the SD of total sleep time), (3) sleep onset (the mean clock time of falling asleep), and (4) sleep offset (the mean clock time of wake-up) [51].

#### Rest-Activity Rhythms

We extracted five nonparametric features from the Fitbit step count recordings to characterize the stability, fragmentation, timing, and mean activity level of participants’ rest-activity rhythms, using the R package *nparACT* (R Foundation for Statistical Computing) [57,58]. These features include (1) intradaily variability of steps (step IV), quantifying the fragmentation in the rest-activity cycle; (2) interdaily stability of steps (step IS), quantifying the stability of rest-activity patterns over a 14-day PHQ-8 interval; (3) L5 onset, representing the onset of least active continuous 5-hour period; (4) M10 onset, representing the onset of the most active continuous 10-hour period; and (5) daily step, representing the mean of daily total steps in a 14-day PHQ-8 interval [57,58].

#### Circadian Rhythm in HR

For estimating circadian rhythms in HR, we used cosinor analysis—fitting a cosine wave to time series behavioral data through least-squares regression, which has been widely used in previous mHealth studies [59-61]. Using the R package *cosinor*, we performed the cosinor analysis on Fitbit HR data of each 14-day PHQ-8 interval and extracted the following parameters: (1) HR MESOR, the midline estimating statistic of the fitted cosine wave for HR; (2) HR amplitude, the difference between the peak value and MESOR of the fitted cosine wave for HR; and (3) HR acrophase, the timing of the HR peak [59-61].

### Statistical Analysis

Given the longitudinal nature of our data set, that is, each participant had repeated measurements, we used the linear mixed-effects model [62] with a participant-specific random intercept in this study, implemented using the R package *lmerTest*. To investigate whether disregarding seasonal effects biases the associations between depression severity and circadian rhythms, we established and compared the following 3 models for each of circadian rhythm features.

- Model 1: A linear mixed-effects model was established to regress each circadian rhythm feature with only the PHQ-8 score as the independent variable.
- Model 2: Season was included as an independent variable in addition to the PHQ-8 score, considering seasonal effects on the circadian rhythms feature.
- Model 3: To further explore potential variations in the association between depression severity and circadian rhythms across seasons, an interaction term between the PHQ-8 score and season was added to the main-effects model (model 2).

All models were adjusted by covariates: age, gender, study site, lockdown, and employment status. The equations of these 3 models are outlined as follows:

- Model 1: Circadian rhythm = β_1_ PHQ‑8 + COVs
- Model 2: Circadian rhythm = β_1_ PHQ‑8 + β_2_ season + COVs
- Model 3: Circadian rhythm = β_1_ PHQ‑8 + β_2_ season + β_3_ PHQ‑8 × season + COVs

where COVs represents all covariates mentioned above and circadian rhythm is one of the wearable-measured circadian rhythm features.

Likelihood ratio tests were then performed to examine whether including more variables (season and the interaction term) can significantly improve the fitting of the regression model. The Benjamini-Hochberg method was used for the correction of multiple comparisons [63]. Despite the inclusion of lockdown as a covariate, we also repeated our analysis on the subset of data before the COVID-19 pandemic (before January 31, 2020).

## Results

### Data Summary

**Figure 2:**
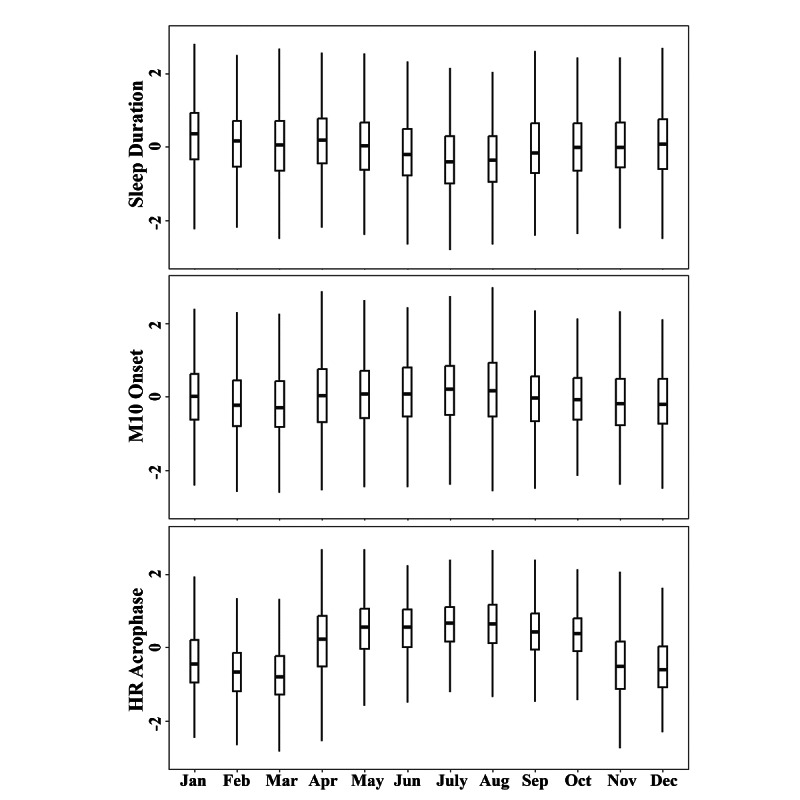
Variations of wearable-measured circadian rhythm features across a year. For each participant, circadian rhythm features were normalized to reduce the individual differences. HR: heart rate.

In accordance with our data inclusion criteria (see the Methods section), we analyzed a total of 10,018 PHQ-8 records alongside corresponding Fitbit data from 543 participants, with an average of 16 recordings per participant. The cohort selected for this study had a median age of 48 (IQR 32-58) years, was predominantly female (n=414, 76.2%), and included 230 (42.4%) employed participants. The distribution of PHQ-8 records was approximately uniform across the seasons, with winter accounting for 26.9%, spring 23.6%, summer 25.5%, and autumn 23.9%. We observed that demographics and data collection varied across study sites, with participants at the CIBER (Centro de Investigación Biomédica en Red) site in Spain being older and more likely to be retired, whereas the King’s College London site in the United Kingdom had the highest number of participants and PHQ-8 records collected. Table 1 summarizes participant demographics and PHQ-8 records for the entire cohort, with site-specific comparisons. Figure 2 visualizes the variations of wearable-measured circadian rhythm features across a year.

**Table 1:** A summary of demographics of participants and PHQ-8^a^ questionnaires in the present study, with the site-specific comparisons.^b^

Characteristic	Total	CIBER^c^ (Spain)	KCL^d^ (UK)	VUMC^e^ (Netherlands)
Participants, n	543	126	303	114
Age (years), median (IQR)	48.00 (32.00-58.00)	53.00 (47.25-60.00)	44.00 (30.00-56.00)	39.50 (26.00-57.75)
Female, n (%)	414 (76.2)	91 (72.2)	233 (76.9)	90 (78.9)
Employed, n (%)	230 (42.4)	30 (23.8)	164 (54.1)	36 (31.6)
PHQ-8 records, n	10,018	1990	5899	2129
Records in winter, n (%)	2699 (26.9)	541 (27.2)	1519 (25.8)	639 (30.0)
Records in spring, n (%)	2363 (23.6)	450 (22.6)	1504 (25.5)	409 (19.2)
Records in summer, n (%)	2559 (25.5)	520 (26.1)	1508 (25.6)	531 (24.9)
Records in autumn, n (%)	2397 (23.9)	479 (24.1)	1368 (23.2)	550 (25.8)
Participants before the COVID-19 pandemic, n	442	115	259	68
Records before the COVID-19 pandemic, n (%)	4202 (41.9)	999 (50.2)	2650 (44.9)	553 (26.0)

^a^PHQ-8: 8-item Patient Health Questionnaire.

^b^Due to the private issue, participants’ specific geographic information was not collected.

^c^CIBER: Centro de Investigación Biomédica en Red.

^d^KCL: King’s College London.

^e^VUMC: Vrije Universiteit Medisch Centrum.

### Associations Between the PHQ-8 score and Wearable-Measured Circadian Rhythm Features

The results of models 1 and 2 are displayed in Table 2. The likelihood ratio tests revealed that incorporating seasonal effects (model 2) significantly improved the model fit for all 12 circadian rhythm features. Specifically, model 2 demonstrated significant positive associations of the PHQ-8 score with several features: sleep duration (β_1_=0.46, *P*<.001), sleep onset (β_1_=0.55, *P*=.001), sleep offset (β_1_=1.12, *P*<.001), sleep variability (β_1_=0.96, *P*<.001), L5 onset (β_1_=0.43, *P*=.04), M10 onset (β_1_=0.73, *P*=.003), and HR acrophase (β_1_=0.71, *P*=.001). Conversely, significant negative associations were observed with daily step (β_1_=–93.61, *P*<.001), step IS (β_1_=–0.001, *P*<.001), HR MESOR (β_1_=–0.03, *P*<.001), and HR amplitude (β_1_=–0.04, *P*<.001). However, the effect sizes of PHQ-8 on step IS, HR MESOR, and HR amplitude are small, according to their practical meanings.

**Table 2:** Effects of PHQ-8^a^ and seasonality on wearable-measured circadian rhythm features estimated by mixed-effects regression models (models 1^b^ and 2^c^).^d^

Feature	Model 1	Model 2					Likelihood ratio test (*P* values)
	PHQ-8, β_1_ (SE)	PHQ-8, β_1_ (SE)	Spring, β_2_ (SE)	Summer, β_2_ (SE)	Autumn, β_2_ (SE)		
Sleep duration	0.51 (0.14)***	0.46 (0.14)***	–6.48 (1.32)***	–16.63 (1.31)***	–6.13 (1.31)***	<.001	
Sleep onset	0.52 (0.17)**	0.55 (0.17)**	3.68 (1.61)*	7.16 (1.59)***	–4.64 (1.60)**	<.001	
Sleep offset	1.13 (0.15)***	1.12 (0.15)***	–0.25 (1.44)	–6.91 (1.43)***	–8.32 (1.43)***	<.001	
Sleep variability	0.97 (0.14)***	0.96 (0.14)***	–8.48 (1.36)***	–5.29 (1.35)***	–2.82 (1.35)*	<.001	
Daily step	–94.71 (6.43)***	–93.61 (6.41)***	–19.21 (61.28)	394.46 (60.81)***	186.16 (60.75)**	<.001	
Step IV^e^	0.001 (0.0006)	0.0007 (0.0006)	0.001 (0.006)	–0.026 (0.006)***	–0.021 (0.006)***	.006	
Step IS^f^	–0.001 (0.0002)***	–0.001 (0.0002)***	0.002 (0.002)	–0.006 (0.002)*	0.004 (0.002)	.007	
L5 onset^g^	0.43 (0.21)*	0.43 (0.21)*	–1.10 (2.13)	–3.84 (2.12)	–3.98 (2.12)	.002	
M10 onset^h^	0.66 (0.25)**	0.73 (0.25)**	11.94 (2.45)***	20.51 (2.43)***	1.61 (2.44)	<.001	
HR^i^ MESOR^j^	–0.03 (0.01)***	–0.03 (0.01)***	–0.47 (0.09)***	–0.01 (0.09)	0.04 (0.09)	<.001	
HR amplitude	–0.04 (0.01)***	–0.04 (0.01)***	0.19 (0.05)***	0.92 (0.05)***	0.39 (0.05)***	<.001	
HR acrophase	0.51 (0.24)*	0.71 (0.22)**	43.35 (2.15)***	67.94 (2.13)***	24.09 (2.13)***	<.001	

^a^PHQ-8: 8-item Patient Health Questionnaire.

^b^Model 1: Circadian rhythm = β_1_ PHQ‑8 + COVs.

^c^Model 2: Circadian rhythm = β_1_ PHQ‑8 + β_2_ season + COVs, where COVs represents covariates mentioned in the Methods section. Note that winter is the reference season.

^d^The exact *P* values are reported in the main text.

^e^IV: intradaily variability.

^f^IS: interdaily stability.

^g^L5 onset: the onset of least active continuous 5-hour period.

^h^M10 onset: the onset of the most active continuous 10-hour period.

^i^HR: heart rate.

^j^HR MESOR: midline estimating statistic of the fitted cosine wave for heart rate.

*P<.05, **P<.01, ***P<.001.

The results of model 3, along with the likelihood ratio comparison with model 2, are summarized in Table 3. We found that the inclusion of the interaction term between PHQ-8 and seasonality significantly improved the model fit for daily step, step IV, step IS, M10 onset, HR MESOR, and HR amplitude. Notably, the negative association between the PHQ-8 score and daily step exhibited greater strength in spring (β_3_ of PHQ-8 × spring = –31.51, *P*=.002) and summer (β_3_ of PHQ-8 × summer = –42.61, *P*<.001) than in winter. Additionally, a significant positive association between M10 onset and PHQ-8 was observed exclusively in summer (β_3_ of PHQ-8 × summer = 1.06, *P*=.008), with no significant associations observed in other seasons.

**Table 3:** Coefficients, SE, and significance^a^ of PHQ-8^b^, seasonality, and the interaction term between PHQ-8 and seasons in model 3^c^ for the entire data set.

Feature	PHQ-8, β_1_ (SE)	Spring, β_2_ (SE)	Summer, β_2_ (SE)	Autumn, β_2_ (SE)	PHQ-8 × spring β_3_ (SE)	PHQ-8 × summer, β_3_ (SE)	PHQ-8 × autumn, β_3_ (SE)	Likelihood ratio test (*P* values)
Sleep duration	0.49 (0.18)**	–4.55 (2.49)	–16.27 (2.45)***	–7.2 (2.46)**	–0.20 (0.22)	–0.04 (0.22)	0.11 (0.21)	.50
Sleep onset	0.79 (0.22)***	4.57 (3.04)	11.14 (2.98)***	0.55 (3)	–0.09 (0.26)	–0.41 (0.26)	–0.53 (0.26)*	.49
Sleep offset	1.01 (0.20)***	–2.88 (2.71)	–7.28 (2.67)**	–9.74 (2.68)***	0.27 (0.24)	0.04 (0.23)	0.15 (0.23)	.53
Sleep variability	1.23 (0.19)***	–4.32 (2.56)	0.19 (2.52)	–1.43 (2.53)	–0.42 (0.22)	–0.57 (0.22)**	–0.14 (0.22)	.26
Daily step	–73.21 (8.55)***	289.04 (115.3)*	804.05 (113.32)***	311.21 (113.92)**	–31.51 (10.01)**	–42.61 (9.95)***	–12.66 (9.87)	<.001
Step IV^d^	–0.001 (0.0008)	–0.02 (0.01)	–0.06 (0.01)***	–0.04 (0.01)***	0.002 (0.001)*	0.004 (0.001)***	0.002 (0.001)	<.001
Step IS^e^	–0.001 (0.0003)**	0.01 (0.005)*	–0.001 (0.004)	0.005 (0.004)	–0.001 (0.0004)*	–0.001 (0.0004)	–0.0001 (0.0004)	<.001
L5 onset^f^	0.51 (0.29)	–4.11 (4.02)	1.26 (3.95)	–2.89 (3.98)	0.31 (0.35)	–0.54 (0.35)	–0.11 (0.34)	.23
M10 onset^g^	0.26 (0.34)	4.86 (4.63)	10.33 (4.54)*	–0.44 (4.57)	0.72 (0.41)	1.06 (0.40)**	0.21 (0.40)	.04
HR^h^ MESOR^i^	–0.03 (0.01)*	–0.57 (0.16)***	0.05 (0.16)	0.28 (0.16)	0.01 (0.01)	–0.01 (0.01)	–0.03 (0.01)	.003
HR amplitude	–0.04 (0.01)***	0.36 (0.1)***	0.87 (0.1)***	0.38 (0.1)***	–0.02 (0.01)*	0.01 (0.01)	0.002 (0.008)	.001
HR acrophase	0.50 (0.30)	40.39 (4.05)***	62.99 (3.98)***	23.3 (4)***	0.30 (0.35)	0.52 (0.35)	0.08 (0.35)	.75

^a^The exact *P* values are reported in the main text.

^b^PHQ-8: 8-item Patient Health Questionnaire.

^c^Model 3: Circadian rhythm = β_1_ PHQ‑8 + β_2_ season + β_3_ PHQ‑8 × season + COVs, where COVs represents covariates mentioned in the Methods section. Note that winter is the reference season.

^d^IV: intradaily variability.

^e^IS: interdaily stability.

^f^L5 onset: the onset of least active continuous 5-hour period.

^g^M10 onset: the onset of the most active continuous 10-hour period.

^h^HR: heart rate.

^i^HR MESOR: midline estimating statistic of the fitted cosine wave for heart rate.

*P<.05, **P<.01, ***P<.001.

### Seasonal Effects on Wearable-Measured Circadian Rhythm Features

Our results reveal significant seasonal impacts on various circadian rhythms, as indicated by the seasonal coefficients in model 2 (Table 2). Notably, HR acrophase varied significantly across seasons, delayed by 43.4 minutes in spring (*P*<.001), 67.9 minutes in summer (*P*<.001), and 24.1 minutes in autumn (*P*<.001) compared with winter (the reference season).

In terms of rest-activity rhythms, compared with winter, summer was associated with 394.5 more daily steps (*P*<.001) and 0.03 lower step IV (*P*<.001), and M10 onset was 20.5 minutes later (*P*<.001); autumn was associated with 186.2 more daily steps (*P*=.002) and 0.02 lower step IV (*P*<.001); and spring was associated M10 onset being 11.9 minutes later (*P*<.001).

Regarding sleep-wake rhythms, compared with winter, we found that (1) sleep duration decreased by 6.5 minutes in spring (*P*<.001), 16.6 minutes in summer (*P*<.001), and 6.1 minutes in autumn (*P*<.001); (2) sleep onset was 4.6 minutes earlier in autumn (*P*=.004), 3.7 minutes later in spring (*P*=0.02), and 7.2 minutes later in summer (*P*<.001); (3) sleep offset was 6.9 minutes earlier in summer (*P*<.001) and 8.3 minutes earlier in autumn (*P*<.001); and (4) sleep variability was 8.5 minutes lower in spring (*P*<.001), 5.3 minutes lower in summer (*P*<.001), and 2.8 minutes lower in autumn (*P*=.04).

### Site-to-Site Analysis

Results of our subset analyses across 3 study sites are shown in Tables S1-S3 in Multimedia Appendix 1. Despite a reduction in sample sizes leading to some nonsignificant coefficients, the overall direction of associations between the PHQ-8 and circadian rhythm features remained consistent with the results in the entire data set. Notably, the seasonal changes in sleep-related features and HR acrophase are larger in the Spain site than the sites in the United Kingdom and Netherlands. For instance, during summer compared with winter, participants from the Spain site experienced a significant decrease in sleep duration by 36.6 minutes, whereas participants from the sites in the United Kingdom and Netherlands experienced reductions in sleep duration by 18.9 and 8.5 minutes, respectively. Furthermore, compared with winter, the HR acrophase for participants in Spain was delayed by 123.2 minutes in summer, significantly more than the delays of 58.2 minutes in the UK site and 64.0 minutes in the Netherlands site during summer.

### Pre–COVID-19 Subset Analysis

The pre–COVID-19 subset analysis (Table 4) indicated that the directions and significances of associations between depression severity and circadian rhythm features were consistent with results in the entire data set. The seasonal effects on most circadian rhythm features (except for daily step) were similar between the entire data set and the pre–COVID-19 subset. Notably, daily step exhibited larger seasonal fluctuations in the pre–COVID-19 period. Specifically, compared with winter, participants in the pre–COVID-19 subset exhibited 657.2, 1096.4, and 321.6 more daily steps in spring, summer, and autumn, respectively.

**Table 4:** Coefficients, SE, and significance^a^ of PHQ-8^b^, seasonality, and the interaction term between PHQ-8 and seasons in model 3^c^ for the pre-COVID subset.

Feature	PHQ-8, β_1_ (SE)	Spring, β_2_ (SE)	Summer, β_2_ (SE)	Autumn, β_2_ (SE)	PHQ-8 × spring, β_3_ (SE)	PHQ-8 × summer, β_3_ (SE)	PHQ-8 × autumn, β_3_ (SE)
Sleep duration	0.97 (0.26)***	–3.01 (3.99)	–14.86 (3.67)***	–6.88 (3.45)*	–0.01 (0.34)	–0.05 (0.31)	0.11 (0.29)
Sleep onset	0.87 (0.36)*	–2.1 (5.46)	9.84 (5.02)	0.58 (4.73)	–0.25 (0.46)	–0.51 (0.43)	–0.64 (0.39)
Sleep offset	1.43 (0.31)***	–6.17 (4.65)	–7.6 (4.27)	–10.04 (4.03)*	–0.24 (0.39)	–0.22 (0.36)	0.07 (0.34)
Sleep variability	1.47 (0.26)***	1.94 (4.09)	2.29 (3.76)	0.62 (3.55)	–0.76 (0.35)*	–0.89 (0.32)**	–0.33 (0.3)
Daily step	–71.62 (12.05)***	657.17 (179.07)***	1096.36 (164.67)***	321.63 (155.06)*	–20.45 (15.21)	–48.65 (13.99)***	1.49 (12.91)
Step IV^d^	–0.001 (0.001)	–0.04 (0.02)*	–0.03 (0.02)	–0.01 (0.02)	0.004 (0.002)*	0.003 (0.001)*	0.0007 (0.0013)
Step IS^e^	–0.0003 (0.0005)	–0.004 (0.0072)	0.0021 (0.0066)	0.0035 (0.0063)	–0.0003 (0.0006)	–0.0007 (0.0006)	–0.0002 (0.0005)
L5 onset^f^	0.57 (0.45)	–17.84 (7.16)*	–6.74 (6.58)	–2.26 (6.22)	0.4 (0.61)	–0.2 (0.56)	–0.17 (0.52)
M10 onset^g^	0.09 (0.51)	–7.37 (7.89)	4.83 (7.25)	1.28 (6.84)	1.14 (0.67)	1.22 (0.62)*	0.07 (0.57)
HR^h^ MESOR^i^	–0.08 (0.02)***	–0.4 (0.27)	–0.53 (0.25)*	–0.38 (0.23)	0.01 (0.02)	0.04 (0.02)*	0.04 (0.02)*
HR amplitude	–0.05 (0.01)***	0.31 (0.17)	0.58 (0.15)***	0.07 (0.14)	–0.01 (0.01)	0.02 (0.01)	0.02 (0.01)
HR acrophase	0.52 (0.38)	35.11 (5.77)***	68.14 (5.3)***	22.2 (5.0)***	1.15 (0.49)*	0.26 (0.45)	0.19 (0.42)

^a^The exact *P* values are reported in the main text.

^b^PHQ-8: 8-item Patient Health Questionnaire.

^c^Model 3: Circadian rhythm = β_1_ PHQ‑8 + β_2_ season + β_3_ PHQ‑8 × season + COVs, where COVs represents covariates mentioned in the Methods section.

^d^IV: intradaily variability.

^e^IS: interdaily stability.

^f^L5 onset: the onset of least active continuous 5-hour period.

^g^M10 onset: the onset of the most active continuous 10-hour period.

^h^HR: heart rate.

^i^HR MESOR: midline estimating statistic of the fitted cosine wave for heart rate.

*P<.05, **P<.01, ***P<.001.

## Discussion

### Primary Findings

Our study, derived from a large European longitudinal mHealth study focused on depression, revealed significant seasonal effects on wearable-measured circadian rhythms and their associations with depression.

One of our key findings is that the associations between depression severity and certain wearable-measured circadian rhythms varied across different seasons. Specifically, we found a stronger negative association between depression and daily step count during the warmer seasons of spring and summer compared with winter. Additionally, a significant positive association was identified between depression severity and the onset of the most active continuous 10-hour period exclusively in summer. This may be attributed to the more favorable weather conditions for outdoor activities in summer [64], which could increase the observable relationship between rest-activity rhythms and depression. Furthermore, we found that the inclusion of seasonal effects can significantly improve the model fit for all wearable-measured circadian rhythms. These findings highlight the critical importance of including seasonal effects in longitudinal mHealth studies.

On adjusting for seasonal effects, we found that higher depression severity was significantly associated with lower levels of physical activity (daily step), more irregular activities (sleep variability and step IS), and later circadian rhythm timings (sleep onset and offset, M10 onset, and HR acrophase). These relationships are consistent with prior research. The linkage between reduced physical activity levels and worsened depression severity was reported in both survey-based and mHealth studies [65,66]. The preventive and therapeutic roles of physical activity against depression have been well-documented [67,68]. Moreover, there is extensive evidence linking depression with more irregular daily behaviors, including increased sleep variability [51,69-71], less stable rest-activity rhythms [72], and irregular patterns in Bluetooth [52] and GPS [73] data. The delayed circadian rhythm timings have been found to be associated with higher depression severity in multiple data streams, such as sleep recordings [51,69-71], activity logs [61], and HR recordings [74]. These consistent findings highlight the close associations between circadian rhythms and depression severity.

This study also revealed significant seasonal impacts on circadian rhythms, most notably between summer and winter. The cohort demonstrated shorter and later sleep patterns, increased daily step counts, reduced intradaily step variability, and delayed circadian rhythm timings during summer compared with winter. Especially, the circadian phase (HR acrophase) was delayed by 67.9 minutes in summer. These findings mostly align with previous laboratory and survey-based studies, which suggested that people tend to sleep longer in winter, possibly due to the effects of light exposure on melatonin production [75-78]. Previous studies have similarly observed higher activity levels [36,38,39], lower intradaily variability [79,80], and delayed hormone secretion rhythm [81,82] in summer compared with winter, further supporting our findings.

Conducted on a large cohort over an extended period using 3 wearable data modalities, this study aligned with some previous survey and laboratory findings, highlighting the precision of mobile technology in tracking behavioral rhythms. Our findings suggest that wearable-measured circadian rhythms have the potential to be digital biomarkers for depression detection. Furthermore, this study could inform the future design of seasonal, context-sensitive mHealth interventions, such as tailored activity recommendations and light exposure therapy.

### Limitations

This study has several limitations that may impact its findings and interpretations. First, the presence of missing data may introduce bias, as our previous study indicated data compliance is associated with depression severity and other personal traits (eg, age) [83]. Second, the specificity of our cohort, characterized by a history of depression and a predominantly female composition, may limit the generalizability of our findings, highlighting the need for validation in more general populations. Third, while we accounted for national lockdowns as a covariate in the regression models and conducted analysis on the pre–COVID-19 subset, the complexities and varied impacts of COVID-19 necessitate additional validations in the postpandemic data sets. Fourth, our study relied on PHQ-8 scores as depression labels, which may introduce subjective biases.

Lastly, while we adjusted for study sites (in different countries) as a covariate in our regression models and conducted the site-to-site analyses, the influence of geographical location on circadian rhythms and their relationship is still needed to be explored in future research, ideally through the randomized controlled trials.

### Conclusions

Our analysis of longitudinal wearable data from a large cohort reveals the significant seasonal impact on circadian rhythms and their associations with depression, emphasizing the importance of accounting for seasonal variations in longitudinal mHealth research. Additionally, we found that wearable-measured circadian rhythms are significantly linked to depression severity, indicating their potential to be digital biomarkers for depression detection. These findings enrich our understanding of the mechanisms and pathology underlying depression and could inform the future design of mental health monitoring and interventions.

## Appendix

Multimedia Appendix 1 Results of the subset analyses across 3 study sites.

## Abbreviations

BRC: Biomedical Research Centre
EFPIA: European Federation of Pharmaceutical Industries and Associations
HR: heart rate
IMI: Innovative Medicines Initiative
mHealth: mobile health
MRC: Medical Research Council
NHS: National Health Service
NIHR: National Institute for Health Research
PHQ-8: 8-item Patient Health Questionnaire
RADAR-MDD: Remote Assessment of Disease and Relapse Major Depressive Disorder
