# Supplementary material for: Longitudinal Assessment of Seasonal Impacts and Depression Associations on Circadian Rhythm Using Multimodal Wearable Sensing: Retrospective Analysis
Source: J Med Internet Res. 2024 Jun 28;26:e55302. doi: 10.2196/55302 (PMC11245656; doi:10.2196/55302)
Supplement: Multimedia Appendix 1 [file jmir_v26i1e55302_app1.docx]

**Table S1.** **Coefficients, standard error (SE), and significance^a^ of PHQ-8, seasonality, and the interaction term between PHQ-8 and seasons in linear mixed-effect models^b^ for the CIBER site in Spain.**

| **Feature** | **PHQ8** $\beta_{1}$(SE) | **Spring** $\beta_{2}$(SE) | **Summer** $\beta_{2}$(SE) | **Autumn** $\beta_{2}$(SE) | **PHQ8**$\boldsymbol{\times}$**Spring** $\beta_{3}$(SE) | **PHQ8**$\boldsymbol{\times}$**Summer** $\beta_{3}$(SE) | **PHQ8**$\boldsymbol{\times}$**Autumn** $\beta_{3}$(SE) |
| --- | --- | --- | --- | --- | --- | --- | --- |
| Sleep Duration | 0.85(0.6) | -2.01(9.69) | -36.66(9.2)*** | -17.62(9.44) | -0.79(0.67) | -0.05(0.64) | 0.32(0.65) |
| Sleep Onset | 1.33(0.71) | 23.57(11.32)* | 38.23(10.74)*** | 11.34(11.01) | -1.28(0.78) | -1.68(0.75)* | -0.97(0.75) |
| Sleep Offset | 1.46(0.56)** | 5.44(8.74) | 6.73(8.3) | -13.13(8.5) | -0.19(0.6) | -0.63(0.58) | 0.68(0.58) |
| Sleep Variability | 1.48(0.58)* | 6.69(9.9) | -6.82(9.38) | 2.47(9.65) | -1.41(0.68)* | -0.68(0.65) | -0.63(0.66) |
| Daily Step | -86.64(21)*** | -254.95(329.56) | 296.25(312.81) | 41.88(320.56) | -3.7(22.71) | -25.33(21.76) | -6.89(21.94) |
| Step IV | 0.002(0.002) | 0.09(0.03)** | 0.03(0.03) | 0.02(0.03) | -0.002(0.002) | 0.001(0.002) | -0.0004(0.002) |
| Step IS | -0.001(0.001) | -0.001(0.01) | -0.04 (0.01)*** | 0.002(0.01) | 0.001(0.001) | 0.002(0.001) | 0.0003(0.001) |
| L5 Onset | -0.08(0.73) | 1.16(12.3) | 8.79(11.65) | -6.28(11.98) | 1.02(0.85) | 0.02(0.81) | 0.72(0.82) |
| M10 Onset | -0.8(0.75) | 8.74(12.39) | 27.86(11.75)* | -6.94(12.06) | 0.88(0.85) | 1.08(0.82) | 1.06(0.83) |
| HR MESOR | -0.05(0.03) | -1.26(0.49)* | -0.81(0.46) | -0.37(0.48) | 0.04(0.03) | 0.08(0.03)* | -0.01(0.03) |
| HR Amplitude | 0.003(0.02) | 0.15(0.28) | 0.81(0.26)** | 0.55(0.27)* | -0.02(0.02) | -0.002(0.02) | -0.002(0.02) |
| HR Acrophase | -0.04(0.67) | 66.04(10.69)*** | 123.18(10.14)*** | 27.73(10.4)** | 0.3(0.74) | -0.89(0.71) | 0.87(0.71) |

^a^* *P* < .05, ** *P* < .01, *** *P* < .001.

^b^Model: $Circadian rhythm=\beta_{1}PHQ8+\beta_{2}season+\beta_{3}PHQ8\times season+COVs$, where COVs represents covariates mentioned in the Methods section.

**Table S2.** **Coefficients, standard error (SE), and significance^a^ of PHQ-8, seasonality, and the interaction term between PHQ-8 and seasons in linear mixed-effect models^b^ for the KCL site in the UK.**

| **Feature** | **PHQ8** $\beta_{1}$(SE) | **Spring** $\beta_{2}$(SE) | **Summer** $\beta_{2}$(SE) | **Autumn** $\beta_{2}$(SE) | **PHQ8**$\boldsymbol{\times}$**Spring** $\beta_{3}$(SE) | **PHQ8**$\boldsymbol{\times}$**Summer** $\beta_{3}$(SE) | **PHQ8**$\boldsymbol{\times}$**Autumn** $\beta_{3}$(SE) |
| --- | --- | --- | --- | --- | --- | --- | --- |
| Sleep Duration | -0.002 (0.22) | -5.83(2.81)* | -18.94(2.83)*** | -7.4(2.82)** | 0.18(0.26) | 0.95(0.27)*** | 0.33(0.27) |
| Sleep Onset | 0.84(0.28)** | -1.8(3.54) | 5.95(3.56) | -3.66(3.56) | 0.41(0.33) | -0.38(0.34) | -0.31(0.34) |
| Sleep Offset | 0.97(0.27)*** | -5.54(3.38) | -12.14(3.41)*** | -10.06(3.4)** | 0.53(0.32) | 0.37(0.33) | 0.01(0.32) |
| Sleep Variability | 0.97(0.22)*** | -7.11(2.82)* | -2.33(2.84) | -4.28(2.84) | 0.01(0.26) | -0.19(0.27) | 0.22(0.27) |
| Daily Step | -65.31(11.35)*** | 392.13(141.54)** | 836.34(142.53)*** | 339.63(142.33)* | -53.51(13.21)*** | -41.75(13.62)** | -21.52(13.47) |
| Step IV | -0.001(0.001) | -0.02(0.02) | -0.05 (0.02)*** | -0.04(0.02)** | 0.002(0.001) | 0.002(0.002) | 0.002(0.001) |
| Step IS | -0.001(0.0004) | 0.01 (0.01)* | 0.001(0.01) | 0.01(0.01) | -0.002(0.001)** | -0.0003(0.001) | -0.001(0.001) |
| L5 Onset | 1.4(0.4)*** | 0.09(5.03) | 1.95(5.06) | 0.9(5.06) | -0.92(0.47)* | -1.35(0.48)** | -0.84(0.48) |
| M10 Onset | 0.96(0.46)* | 6.73(5.89) | 14.25(5.93)* | 4.22(5.93) | 0.2(0.55) | 0.42(0.57) | -0.37(0.56) |
| HR MESOR | -0.03(0.02) | -0.61(0.19)** | -0.05(0.19) | 0.08(0.19) | 0.01(0.02) | -0.01(0.02) | -0.01(0.02) |
| HR Amplitude | -0.05(0.01)*** | 0.33(0.12)** | 0.81(0.12)*** | 0.28(0.12)* | -0.02(0.01) | 0.02(0.01) | 0.01(0.01) |
| HR Acrophase | 1.44(0.41)*** | 40.5(5.18)*** | 58.17(5.21)*** | 26.11(5.2)*** | -0.71(0.48) | -0.37(0.5) | -0.77(0.49) |

^a^* *P* < .05, ** *P* < .01, *** *P* < .001.

^b^Model: $Circadian rhythm=\beta_{1}PHQ8+\beta_{2}season+\beta_{3}PHQ8\times season+COVs$, where COVs represents covariates mentioned in the Methods section.

**Table S3.** **Coefficients, standard error (SE), and significance^a^ of PHQ-8, seasonality, and the interaction term between PHQ-8 and seasons in linear mixed-effect models^b^ for the VUMC site in Netherlands.**

| **Feature** | **PHQ8** $\beta_{1}$(SE) | **Spring** $\beta_{2}$(SE) | **Summer** $\beta_{2}$(SE) | **Autumn** $\beta_{2}$(SE) | **PHQ8**$\boldsymbol{\times}$**Spring** $\beta_{3}$(SE) | **PHQ8**$\boldsymbol{\times}$**Summer** $\beta_{3}$(SE) | **PHQ8**$\boldsymbol{\times}$**Autumn** $\beta_{3}$(SE) |
| --- | --- | --- | --- | --- | --- | --- | --- |
| Sleep Duration | 0.68(0.28)* | -7.17(4.25) | -8.49(3.92)* | -4.88(3.98) | -0.05(0.4) | -0.67(0.37) | 0.07(0.36) |
| Sleep Onset | 0.49(0.35) | 13.35(5.25)* | 12.16(4.86)* | 8.18(4.92) | -0.92(0.5) | -0.19(0.45) | -1.27(0.45)** |
| Sleep Offset | 1.07(0.33)** | 1.2(5) | 0.19(4.62) | -3.1(4.69) | -0.4(0.47) | -0.77(0.43) | -0.51(0.43) |
| Sleep Variability | 1.21(0.33)*** | -5.98(4.96) | 6.1(4.58) | 1.21(4.65) | -0.47(0.47) | -0.69(0.43) | -0.32(0.43) |
| Daily Step | -91.14(17.75)*** | 159.72(265.21) | 907.09(245.05)*** | 295.44(248.62) | 33.83(25.14) | -39.48(22.85) | 19.14(22.74) |
| Step IV | -0.002(0.002) | -0.06 (0.03)* | -0.12(0.02)*** | -0.06 (0.02)* | 0.001(0.002) | 0.007(0.002)** | 0.002(0.002) |
| Step IS | -0.002(0.001)** | 0.02 (0.01)* | 0.02(0.01) | 0.0003(0.01) | -0.001(0.001) | -0.002(0.001)* | 0.001(0.001) |
| L5 Onset | -0.38(0.53) | -10.91(7.98) | 6.27(7.36) | -4.77(7.48) | 1.72(0.76)* | -0.72(0.69) | 0.06(0.68) |
| M10 Onset | -0.1(0.68) | 0.87(10.16) | 0.65(9.37) | -5.43(9.52) | 1.66(0.96) | 0.89(0.87) | 0.4(0.87) |
| HR MESOR | 0.01(0.02) | -0.24(0.36) | 1.34(0.34)*** | 1.02(0.34)** | 0.03(0.03) | -0.12(0.03)*** | -0.05(0.03) |
| HR Amplitude | -0.05(0.01)*** | 0.35(0.22) | 0.96(0.2)*** | 0.68(0.2)*** | 0.01(0.02) | -0.0004(0.02) | -0.02(0.02) |
| HR Acrophase | 0.3(0.57) | 40.17(8.57)*** | 63.98(7.92)*** | 22.2(8.03)** | 0.69(0.81) | -0.03(0.74) | 0.001(0.73) |

^a^* *P* < .05, ** *P* < .01, *** *P* < .001.

^b^Model: $Circadian rhythm=\beta_{1}PHQ8+\beta_{2}season+\beta_{3}PHQ8\times season+COVs$, where COVs represents covariates mentioned in the Methods section.
